# Introduction to the Special Issue: Preparation, Physicochemical Properties and Application of Natural Plant Polysaccharides

**DOI:** 10.3390/foods12132457

**Published:** 2023-06-23

**Authors:** Yuge Niu

**Affiliations:** Institute of Food and Nutraceutical Science, School of Agriculture and Biology, Shanghai Jiao Tong University, Shanghai 200240, China; yugeniu@sjtu.edu.cn

As natural products, plant polysaccharides have been demonstrated to induce a variety of biological activities by numerous epidemiological investigations and interventional studies, including immunomodulation and antioxidant, antibacterial, antitumor, hypolipidemic, hypoglycemic processes, etc. [1,2,3] Moreover, its excellent biocompatibility and superior physicochemical properties (e.g., emulsification and gelation properties) enable plant polysaccharides to be widely used in the construction of drug delivery vehicles and the development of novel functional materials, while also acting to improve food [4,5,6,7]. With the increasing prevalence of concepts on healthy living, plant polysaccharides have received increasing attention for their diverse physiological functions and great application potential. The quantity of research-based papers on this topic is rising every year, reflecting this growing interest. These studies often conduct extensive discussions on various issues in the application of plant polysaccharides, such as efficient preparation techniques, detailed structural analysis, and clinical function investigation, providing abundant data to support its application in many fields.

In the preceding years, the analysis of structure–function relationships has become one of the key points in the studies of plant polysaccharides. Extensive studies have demonstrated that structural features such as glycosidic bonding mode, monosaccharide composition, functional group type, and the number and distribution of branched chains can directly affect the functional activities of plant polysaccharides [8,9]. The systematic elaboration of structure–function relationships and clarification of the active structural regions of different polysaccharides can provide guidance for the precise development of more novel products with specific functions. Meanwhile, the complex structure of natural plant polysaccharides leads to a higher requirement for preparation methods, structural characterization and functional activity investigations, thereby promoting the overall development of plant polysaccharide exploration in multiple aspects.

This Special Issue will focus on developments related to the structure and functions of plant polysaccharides and their derivatives, covering preparation and modification, structural elucidation and biological activities. The original published papers elaborated these contents as previously described, providing new insights into the resolution of the structure–function relationships of natural plant polysaccharides, which have positive implications for the further application of polysaccharides in the food field.

A study by Liu et al. [10] introduced the preparation, structural characterization and functional activities of *Houttuynia cordata* polysaccharide (HCP). The authors demonstrated the impact of the structure–function relationship on the antioxidant activity of HCP and highlighted its potential application in glycemic index-lowering foods. Consistent with the above study, Feng et al. [11] used response surface methodology to optimize the extraction process of polysaccharides from *Camellia oleifera* Abel leaves, flowers, seed cakes and fruit shells, as well as to characterize the structural features of the four polysaccharides. Each of them has in vitro antioxidant activities and the potential to be developed as food antioxidants. Li et al. [12] found that the structural differences of two new polysaccharides from *Morchella sextelata* led to variations in their antioxidant capacities, which provided quality material for use in the study of the structure–function relationship of antioxidant activities. The research by Chen et al. [13] highlighted that dextrins in sorghum and oats can exert prebiotic effects without producing glycemic effects, providing more original and supplementary options for the development of special diets. Jing et al. [14] systematically summarized the current status of research on the extraction method, molecular structure and pharmacological activities of the *Coriolus versicolor* polysaccharide (CVP). CVPs possess antioxidant, antitumor and immunomodulatory pharmacological activities, and their functions are closely related to structural properties such as glycosidic bond type and monosaccharide composition. Li et al. [15] focused their attention on the effect of natural polysaccharides on intestinal microbiota in inflammatory bowel disease in order to systematically describe how natural polysaccharides alleviate intestinal inflammation through intestinal microbiota. In terms of future research directions, all reviews mentioned that further analysis of polysaccharides structure–function relationships must be conducted if we are to fully utilize the functional activities of natural polysaccharides and develop more diverse health products.

The diverse functional properties of natural plant polysaccharides warrant more diverse research into the field. In particular, the comprehensive analysis of structure-function relationships will create new changes in the food and pharmaceutical fields. We expect that this special issue will stimulate considerable new research interest and provide inspiration for researchers to promote the development and utilization of natural plant polysaccharides.

## Data Availability

The data presented in this study are openly available in the *Foods* special issue: Preparation, Physicochemical Properties and Application of Natural Plant Polysaccharides, at https://www.mdpi.com/journal/foods/special_issues/WI5JF1386S accessed on 15 June 2023.
